# Metabolic dysregulation in patients with premature ovarian insufficiency revealed by integrated transcriptomic, methylomic and metabolomic analyses

**DOI:** 10.1002/ctm2.1006

**Published:** 2022-10-31

**Authors:** Cuiling Lu, Caimeng Qin, Zheng Fu, Lina Wang, Yanxiao Yi, Mingwei Xin, Xiumei Zhen, Chunsheng Han, Cuiling Lu

**Affiliations:** ^1^ National Laboratory of Biomacromolecules CAS Center for Excellence in Biomacromolecules Institute of Biophysics Chinese Academy of Sciences Beijing China; ^2^ Center for Reproductive Medicine Peking University Third Hospital Beijing China; ^3^ Reproductive Medicine Center of Henan Provincial People's Hospital Zhengzhou China; ^4^ Department of Traditional Chinese Medicine Beijing Obstetrics and Gynecology Hospital Capital Medical University Beijing China; ^5^ Beijing Maternal and Child Health Care Hospital Beijing China; ^6^ National Clinical Research Center for Obstetrics and Gynecology Beijing China; ^7^ State Key Laboratory of Stem Cell and Reproductive Biology Institute of Zoology Chinese Academy of Sciences Beijing China; ^8^ Savaid Medical School University of Chinese Academy of Sciences Beijing China; ^9^ Institute for Stem Cell and Regeneration Chinese Academy of Sciences Beijing China

Dear Editor,

Premature ovarian insufficiency (POI) is a disorder of ovarian function, which occurs in approximately 3.7% of women younger than 40 years of age.^1^ The aetiologies of most cases were unknown and usually manifest as isolated organ senescence at early stage of diagnosis, only with long‐term consequences of increased risk of metabolic syndrome or disease.^2^ We tried to explore the essence of POI from multiple dimensions. First, we found that upregulated genes in POI patients’ ovarian granulosa cells were mainly enriched in metabolic pathways. Then, we profiled metabolites in the patients’ sera and identified some metabolites with changed levels, including fumarate, arachidonate and acetoacetate, and the combination of hyodeoxycholic acid (HDCA) and acetoacetate may be used as one of potential biomarkers for POI. In addition, we found genes with upregulated transcription levels and differential methylated levels were enriched in oxidative stress pathways, which can be also elucidated from abnormal metabolism.The transcriptomes of patient and control granulosa cells were profiled, and 3966 differentially expressed genes (DEGs) (fold change > 2, *P*_adj_ < .05) were identified (Table S1). Gene ontology analyses found that downregulated DEGs were mainly related to extracellular matrix/structure organization and different types of junctions. The upregulated DEGs were enriched in various catabolic/metabolic/biosynthetic/processes (Figure 1A). KEGG (Kyoto Encyclopaedia of Genes and Genomes) analysis showed that cytokine–cytokine receptor interaction, PI3K‐Akt‐signalling pathway and MAPK‐signalling pathway were enriched in the downregulated genes, whereas the metabolic pathway was the most enriched in the upregulated genes. Moreover, other pathways, such as amino acid degradation, synthesis, peroxisome and oxidative phosphorylation, were also enriched in upregulated DEGs (Figure 1B).

**FIGURE 1:**
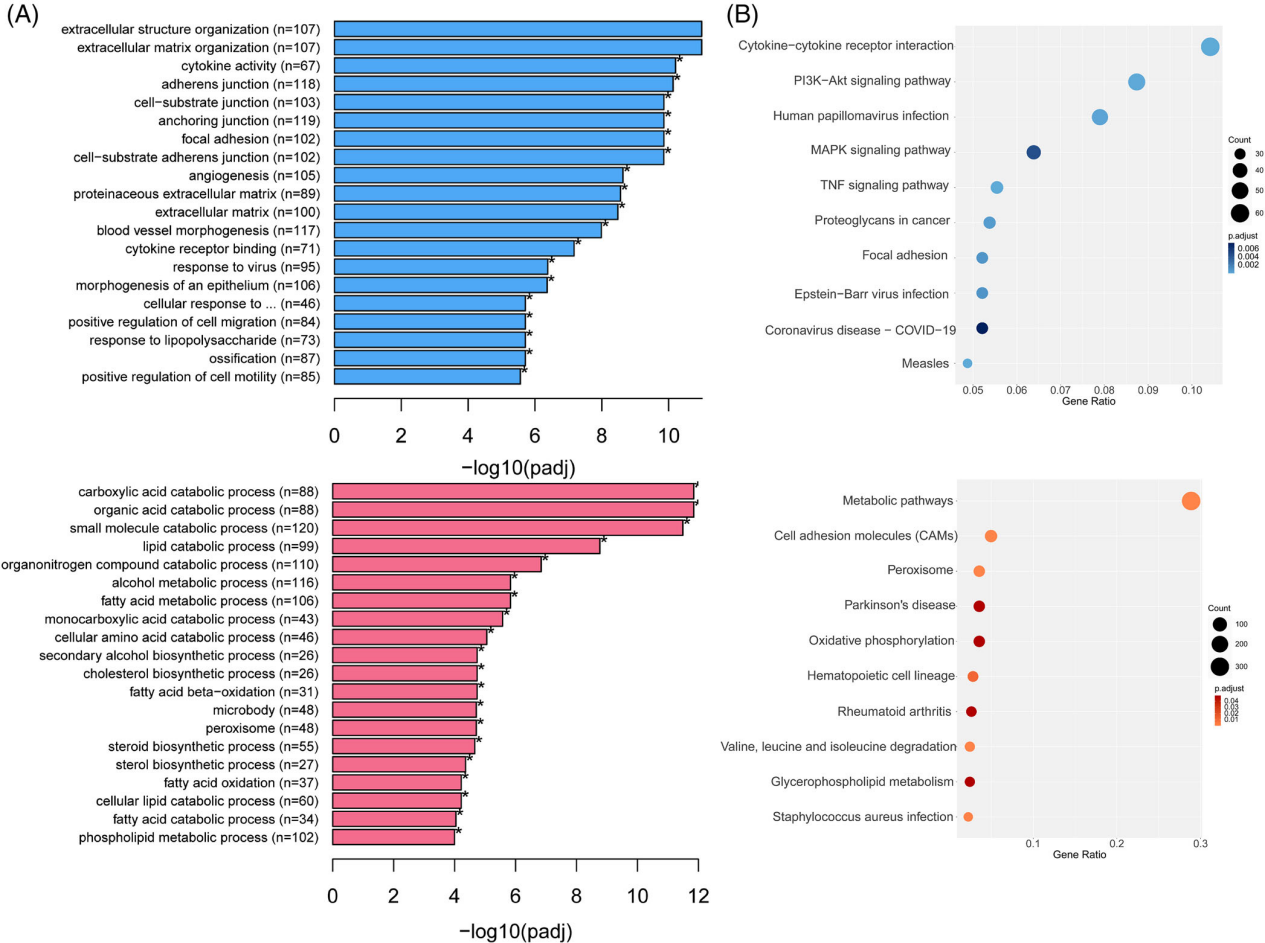
Gene ontology (GO) and Kyoto Encyclopaedia of Genes and Genome (KEGG) analysis of transcriptome of differentially expressed genes (DEGs) between premature ovarian insufficiency (POI) patients and controls. (A) GO enrichment in biological process analysis of DEGs. The *x*‐axis shows −log10 (*p* value), and the *y*‐axis shows the top 20 GO terms in biological process. (B) KEGG enrichment analyses of DEGs. The ratio of the number of DEGs to the total gene number is represented by the enrichment factor. Size of dots: gene number; colour of dots: range of *p*‐values. Red: upregulated DEGs; blue: downregulated DEGs

Several previous studies present valuable information about metabolic features of POI, which mainly focus on total cholesterol, lipoprotein cholesterol and glucose metabolism.^3^, ^4^, ^5^, ^6^ Elevated levels of free fatty acids in follicular fluid were also reported as a potential driver of human POI.^7^ However, many of these results were inconsistent and not so comprehensive, although they have been noteworthy. Therefore, we carried out metabolomic analyses on serum samples from POI patients and controls. The outlined demographic characteristics of all recruited patients and controls were shown (Table 1). Two cohorts were enrolled in the metabolomic analyses in this study (Figure 2A). In the discovery cohort, a total of 6 categories of 181 features were reliably detected. POI patients could be clearly distinguished from controls by conducting multivariate statistical analysis (Figure 2B). Sixty‐two features with VIP (variable importance in projection) values greater than 1 were identified and considered as differential metabolites (DMets). Subsequently, 26 of the 63 DMets were found to be significantly different between POI patients and controls using univariate *t* test analysis (*p* < .05) (Figure 2C; Table S2). Next, 32 out of the 62 features were validated using the validation cohort (Table S3). The numbers of metabolites in the union and intersections of the DMets identified from the discovery and validation cohorts were 39 and 13, respectively. Pearson's correlation coefficient figure and heat map analysis of the 39 DMets are shown (Figure S1). Enrichment and pathway analysis of the 39 DMets are shown respectively (Figure 2D,E). Out of the 13 validated DMets, 5 (acetoacetate, glutamate, C22:1, arachidonate and succinate) and 4 (HDCA, C22:0, homocitrulline [Hcit] and fumarate) were increased and decreased in the sera of POI patients, respectively. In addition, ROS and LDH (lactic dehydrogenase) were increased, and NADH and ATP were decreased in patients (Figure 2F). Receiver‐operating characteristic (ROC) analysis was performed and 8 metabolites had area under curve (AUCs) greater than .7. In addition, the top two combinations with the highest AUCs were NADH plus acetoacetate (.868) and HDCA plus acetoacetate (.955) (Figure 2G,H).

**TABLE 1:** Comparison of the characteristics, endocrine and metabolic parameters among women with premature ovarian insufficiency (POI) and age‐matched controls

	**POI (*n* = 40; 12 for** ^△^)	**CON (*n* = 44; 18 for** ^△^)	***p*‐Value**
Age (years)	31.98 ± 3.87	30.96 ± 3.21	.17
BMI (kg/m^2^)	22.4 ± 3.41	22.25 ± 3.1	.85
AMH (ng/ml)	.08 ± .05	2.44 ± 1.89	<.0001*
FSH (IU/L)	62.48 ± 29.58	6.86 ± 3.9	<.0001*
LH (IU/L)	29.3 ± 26.7	3.8 ± 3.09	<.0001*
PRL (ng/ml)	9.93 ± 9.61	13.85 ± 6.48	.07
E2 (pmol/L)	130.3 ± 115.81	211.41 ± 262.34	.07
T (nmol/L)	.73 ± .14	.95 ± .71	.12
A (nmol/L)	4.71 ± 2.52	7.42 ± 3.37	.001*
P4 (ng/ml)	1.04 ± .58	1.34 ± .81	.06
Glucose (mmol/L)^△^	4.97 ± .39	4.86 ± .33	.41
TC (mmol/L)^△^	4.79 ± .66	4.39 ± .69	.1
TG (mmol/L)^△^	1.05 ± .65	1.02 ± .42	.86

*Note*: Values are the mean ± S.D. ^△^: data from 12 POI and 18 CON.

Abbreviations: A, androstenedione; AMH, anti‐mullerian hormone; BMI, body mass index; E2, estradiol; FSH, follicle‐stimulating hormone; LH, luteinizing hormone; P4, progesterone; POI, premature ovarian insufficiency; PRL, prolactin; T, testosterone; TC, cholesterol; TG, triglyceride.

* indicates statistical significances of *p* < .001.

**FIGURE 2:**
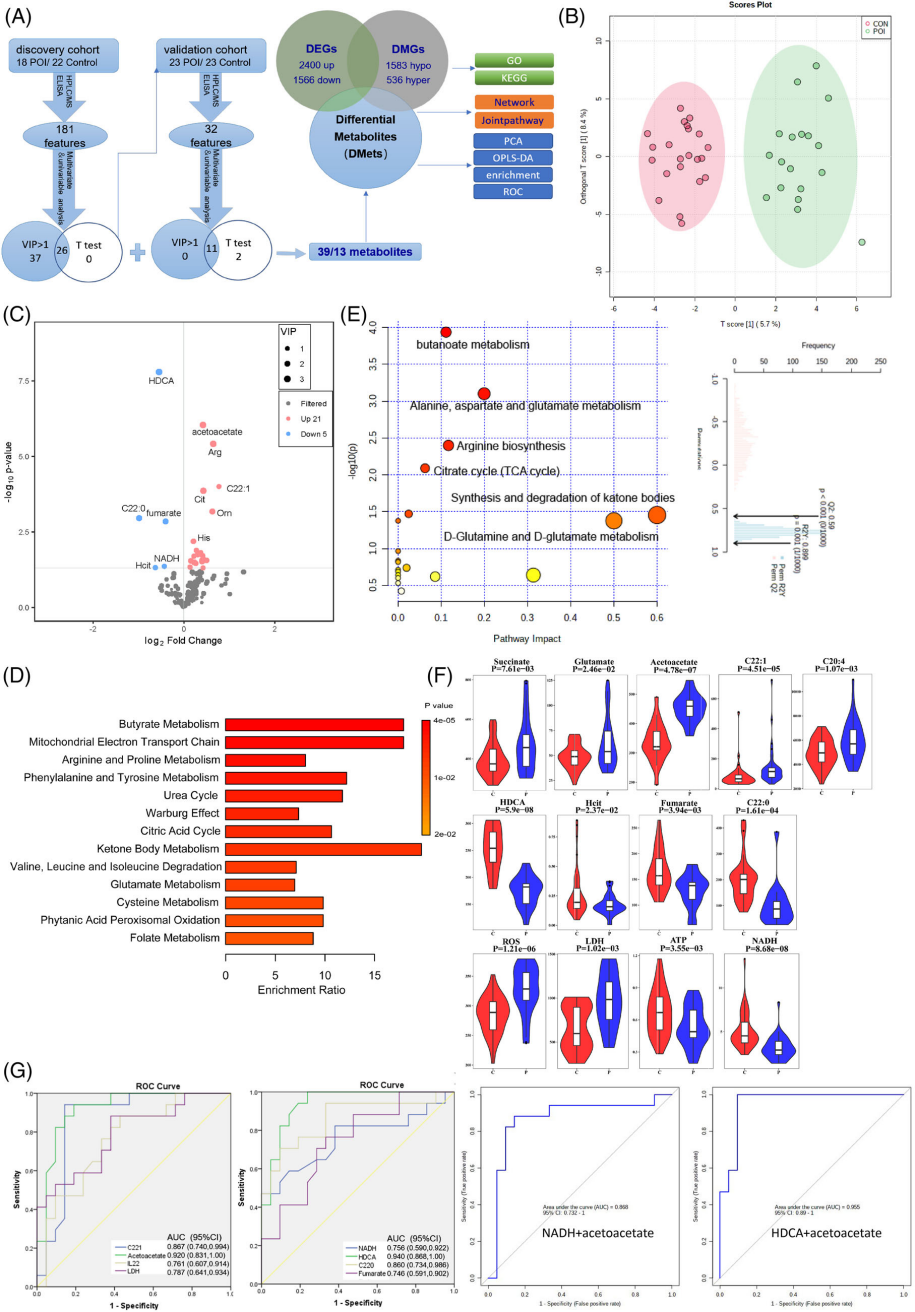
Analysis of metabolites in the cohorts. (A) Flow chart of the study of metabolome and sequential analysis. (B) The OPLS‐DA scatter plots based on the metabolic profiles of two groups in the discovery cohort. Model overview and permutation were also displayed. *R* ^2^ *Y* = .899 and *Q* ^2^ = .59. (C) Volcano plot of univariate statistics and VIP. The threshold value for univariate statistics is *p* < .05; metabolites of variable importance in projection (VIP) scores > 1. Significantly altered metabolites are highlighted in red (increased) and blue (decreased). (D) Enrichment analysis of differential metabolites. (E) Kyoto Encyclopaedia of Genes and Genome (KEGG) pathway of differential metabolites. (F) Violin plots of significantly different metabolites confirmed, red indicates the control group, and blue indicates the premature ovarian insufficiency (POI) group. (G) Receiver operating characteristic (ROC) of potential biomarkers. (A) Potential biomarkers with area under curve (AUC) > .7 (left, increased; right, decreased). (H) The most potential biomarker combinations (left, NADH and acetoacetate; right, hyodeoxycholic acid [HDCA] and acetoacetate)

Furthermore, we reanalysed the human granulosa cell whole‐genome DNA methylation profiling data that we previously generated.^7^ A number of 536 genes were hypermethylated in their differentially methylated regions (DMRs), whereas 1583 genes with hypomethylated DMRs were identified (Table S4; Figure S2). Integrating our transcriptomic and methylomic data, we found that 240 differentially expressed and methylated genes (DEMGs) were mainly enriched in response to oxidative stress, response to chemical stress, reactive oxygen species and hydrogen peroxide (Table S5; Figure S3A,B). *DNMT3, DNMT1*, methylenetetrahydrofolate reductase) and 5‐methyltetrahydrofolate–homocysteine methyltransferase reductase, involved in methionine cycle, were differently expressed in POI patients’ granulosa cells (Figure S3C).

We next conducted network analysis on these DEMGs and the metabolites we detected (Table S6; Figure S4). The top five DMets were ATP, arachidonate, serotonin, glutamate and citrate. Glutamine synthetase (GLUL) and *FOS* were revealed as the top two hub genes in terms of connectivity with metabolites. *GLUL*, which catalyses the ATP‐dependent conversion of glutamate and ammonia to glutamine, is related to 9 metabolites and was significantly upregulated and hypomethylated in exonic regions and the 3′UTRs. In‐line with this, metabolic profiling showed decreased ATP and increased glutamate in POI patients. *FOS* was related to 8 metabolites and significantly upregulated and hypomethylated in exonic and 3′UTR regions (Figure 3A). It was also recently reported as a hub gene in the gene regulatory network of antioxidant in aged monkeys and human ovaries.^8^ In addition, we searched the human metabolome database for the associated enzymes/proteins of the 39 DMets. Changes in the expression and methylation status of these genes were displayed using Cytoscape software (Table S7; Figure S4).

**FIGURE 3:**
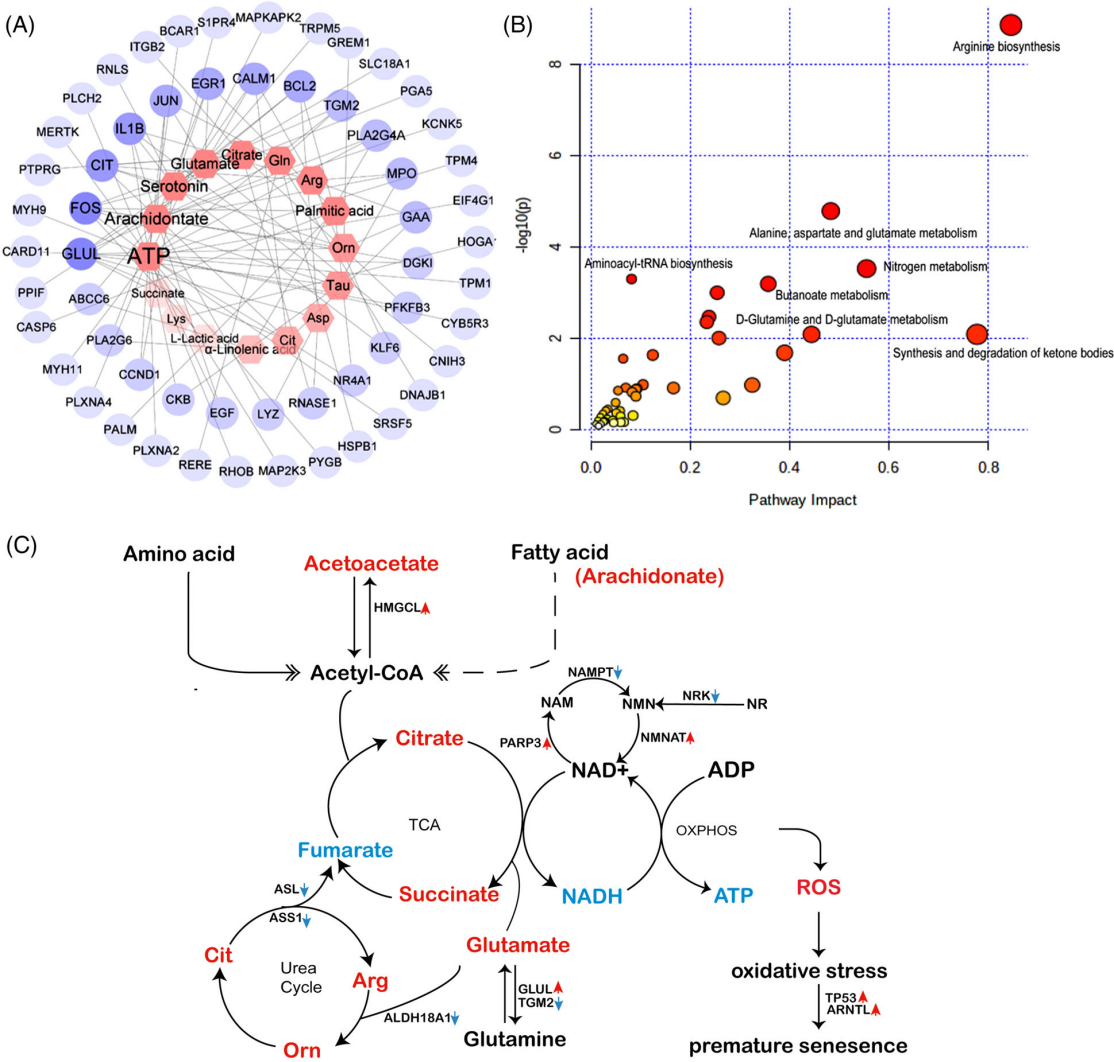
Plot of overlapping differential genes and differential metabolites and schematic summary of abnormal metabolic network. (A) Network analysis of overlapping differentially expressed genes (DEGs) and DMGs genes and 39 metabolites. (B) Joint‐pathway analysis of overlapping genes and 39 metabolites. (C) Schematic diagram of major abnormal metabolic network. Metabolites increased or decreased are denoted by red or blue colour, respectively. The red or blue arrows denote metabolic enzymes that were upregulated or downregulated respectively.

Joint‐pathway analysis of DEMGs and DMets showed pathways those were impacted (Figure 3B). All the previous analyses were summarized as a metabolic network composed of DMets and enzymes of DEMGs that may contribute to the aetiology of POI (Figure 3C). Genes for NMNAT, PAR3 and NAMPT, enzymes involved in the synthesis or consumption of nicotinamide adenine dinucleotide (NAD+), which were reported associated with POI or ovarian ageing,^9^, ^10^ were upregulated or downregulated DEGs.

In summary, the integrated analyses of transcriptomic and methylomic data from granulosa cells of the human ovary and the metabolomic data from human sera have identified increased levels of acetoacetate and arachidonate and disturbances in the TCA (tricarboxylic acid) cycle, fatty acids, ketone bodies and antioxidative processes, all of which contribute to decreased levels of NADH and ATP, increased oxidative stress and eventually ovarian premature senescence. Our data not only yielded valuable fundamental insights into its pathological mechanism but also would aid in the development of new therapeutic targets for POI. The causal effect relationship between gene expression and metabolic changes warrants further investigation in the future.

## CONFLICT OF INTEREST

The authors declare that there is no conflict of interest.

## Supporting information

Supporting Information

Supporting Information

Supporting Information

Supporting Information

Supporting Information

Supporting Information

Supporting Information

Supporting Information

Supporting Information

Supporting Information

Supporting Information

Supporting Information
