# Supplementary material for: Metabolic dysregulation in patients with premature ovarian insufficiency revealed by integrated transcriptomic, methylomic and metabolomic analyses
Source: Clin Transl Med. 2022 Oct 31;12(10):e1006. doi: 10.1002/ctm2.1006 (PMC9619222; doi:10.1002/ctm2.1006)
Supplement: Supplementary file 1 — Supporting Information [file CTM2-12-e1006-s012.docx]

**Materials and Methods**

**Subjects**

All the recruited subjects were from the Reproductive Medical Center of the Third Hospital Peking University (Beijing, China), including two groups of women: POI patients and normal ovarian reserve patients with male factor infertility during assisted reproductive technology. This study investigated the transcriptome granulosa cells from 3 POI patients and 3 controls, and the Methylome of granulosa cells from another 3 POI patients and 3 controls. We also investigated the serum metabolomic profiles of additional 41 POI patients and 45 controls. They were all under 38 years old and had a normal BMI. The diagnoses of POI were based on European Society of Human Reproduction and Embryology 2016 diagnostic criteria. Briefly, the inclusion criteria for POI participation were as follows: (1) oligo/amenorrhea for at least 4 months; (2) an elevated FSH > 25 IU/L on two occasions >4 weeks apart; and (3) chromosome karyotype 46 XX. The criteria for control subjects were as follows: (1) age-matched with the POI group, (2) normal menstrual cycles, and (3) normal hormone levels. Women with histories of anticancer treatment, pelvic surgery, ovarian infection, and/or autoimmune disease were excluded from the study. This study was approved by the Ethics Committee of Peking University Third Hospital (No. IRB00006761-m2020179). Each participant provided written informed consent and received no financial compensation.

**Collection of blood and granulosa cells**

Blood samples were collected by venepuncture after a 12-hour fast. Serum was centrifuged from the blood samples and immediately stored at −80 °C for further detection. Serum concentrations of glucose, lipid, etc. data were derived from the clinical lab. In both groups, the follicular fluid obtained from each patient was collected and centrifuged at 500g for 10 min. The sediment samples were collected for granulosa cell isolation, washed in phosphate-buffered saline (PBS) and centrifuged over Ficoll (GE Healthcare Corp., USA) to remove the red blood cells. Granulosa cells were washed again with PBS, and the cell deposits were flash frozen in liquid nitrogen within 30 min and stored at -80 °C until RNA extraction or DNA extraction.

**RNA-Seq analysis**

Briefly, deposits of granulosa cells were transferred into lysis buffer. The cDNA library was diluted to a final concentration of 2 nM, and a total of 10 pmol of each library was sequenced using an Illumina HiSeq 2000. Six to ten Gb clean data were generated per sample and were used for downstream analyses. Clean data were aligned to the human (hg19) genome using TopHat2 (v2.1.0) with default settings and filtered for uniquely mapped reads. Gene expression values were calculated as FPKM（fragments per kilo base of exon per million fragments mapped）using Cufflinks (v2.2.1). Differential expression analysis of the two groups was performed using the DEGSeq R package (v1.18.0). DEGSeq analysis was used to provide statistical routines for determining differentially expressed genes (DEGs) using a model based on the negative binomial distribution. P values were adjusted using the Benjamini–Hochberg method to control the false discovery rate. Genes with a fold change (FC) >2 and adjusted P value < 0.05 were defined as differentially expressed genes (DEGs). Gene Ontology (GO) enrichment analysis was performed using the GOseq R package with correction for gene length bias. Enrichment of DEGs was conducted using the Kyoto Encyclopedia of Genes and Genomes. GO terms with corrected P values less than 0.05 were considered to be significantly enriched. The enrichment of differentially expressed genes in KEGG pathways was analysed using KOBAS software.

**Methylated DNA data analysis**

We reanalysed the methylated data previously sequenced by whole-genome bisulfite sequencing. Simply, Illumina Hiseq 2500 at Annoroad Gene Technology Co., Ltd (Beijing, China), and 150-bp paired-end reads were generated. GO and KEGG analysis of DMGs (differentially methylated genes) and combined analysis with DEGs were similar to the details described in the RNA-seq data analysis. DMRs between two groups were identified with the R/Bioconductor package eDMR.V.0.5.1, which uses logistic regression to calculate p-values. The p-values were adjusted to q-values using the Sliding Linear Model (SLIM) method. The parameters used for eDMR included a 150 bp window and a 50 bp step size and the DMR were generated with a q-value threshold of 0.05 and a mean methylation difference more than 20%.

**Metabolomics profiling**

High-performance liquid chromatography quadrupole ion trap tandem mass spectrometry (HPLC/Q-TRAP-MS/MS) (Beijing Mass Spectrometry Medical Research Co., and Metabo-profile corporation) was used to detect the content of serum compounds. The HPLC system consisted of an SRD-3600 solvent rack with an analytical 6-channel vacuum degasser, a DGP-3600A pump, a WPS-3000TSL analytical autosampler, and a tcc-3200 column compartment. Chromatographic separation was carried out by an MSLab-AA-C18 column (150 mm × 4.6 mm, 5μm). Analytes were eluted from the column with a gradient using water (containing 0.1% formic acid) (A) and acetonitrile (containing 0.1% formic acid) (B) as the mobile phase. The column temperature was maintained at 50 °C, and the injection volume was 5 μl, with a 1 mL/min flow rate. The main parameters of the mass spectra are the ESI ion source, MRM scanning mode, 5.5 kV IS, and 500 TEM. Nitrogen gas was used as the collision gas in the multiple reaction monitoring (SRM) mode.

**Metabolomics data processing and statistical analysis**

Multivariate statistical analysis, including principal component analysis (PCA) and orthogonal partial least squares discriminant analysis (OPLS-DA), was conducted on the MetaboAnalyst 5.0 website (https://www.metaboanalyst.ca/). OPLS-DA, which is a supervised pattern recognition method, was performed to maximize the variation between groups and to determine the variables contributing to this variation. In the OPLS-DA model, the values of R2 and Q2 were used to assess the goodness of fit and predictive capacity. We performed a permutation test to evaluate it and the model is deemed valid when all Q2 and R2 values to the left are lower than the original points to the right. The variable important in projection (VIP) value of each feature ≥1.0 was used as the common screening criterion for differential metabolites. Then, univariate Student's t test or Mann–Whitney U test were performed after the normal distribution test, and the P value was used to control the error detection rate.

Through multivariate and univariable analyses, the significant differential metabolites were identified by database search, including the Human Metabolome Database (http://[www.hmdb.ca](http://www.hmdb.ca)) and KEGG (http://www.kegg.com). Heatmaps and cluster analysis were conducted with TBtools. Network analysis and pathway analysis were conducted on the MetaboAnalyst website (<https://www.metaboanalyst.ca/>). Receiver operating characteristic (ROC) curve analysis was conducted with IBM SPSS Statistics 20 software. Additionally, a penalized least absolute shrinkage and selection operator (LASSO) logistic regression model analysis (with penalty parameter tuning conducted by 10-fold cross-validation) was performed. Correlation data visualization was realized by Cytoscape software (version 3.8.2).
