# Supplementary figures and images for: Metabolic dysregulation in patients with premature ovarian insufficiency revealed by integrated transcriptomic, methylomic and metabolomic analyses
Source: Clin Transl Med. 2022 Oct 31;12(10):e1006. doi: 10.1002/ctm2.1006 (PMC9619222; doi:10.1002/ctm2.1006)

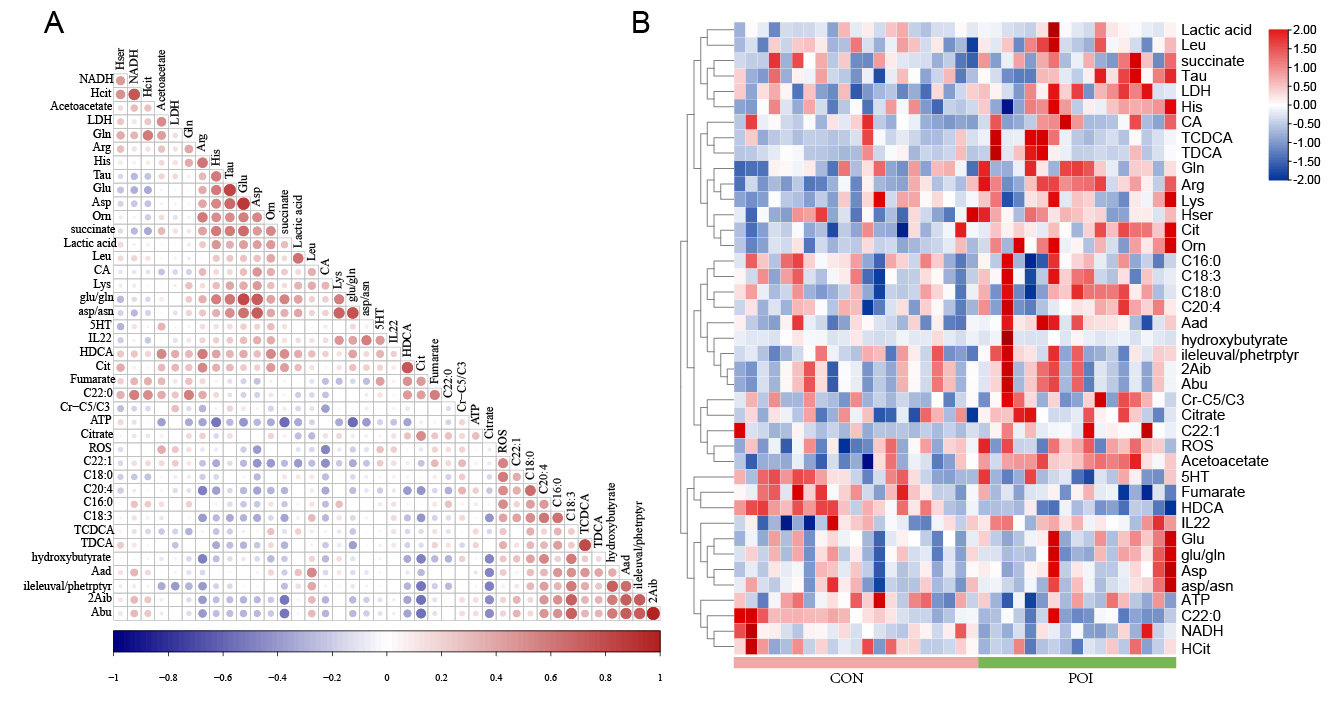

Supplement: Supplementary file 9 — Supporting Information [file CTM2-12-e1006-s009.jpg]

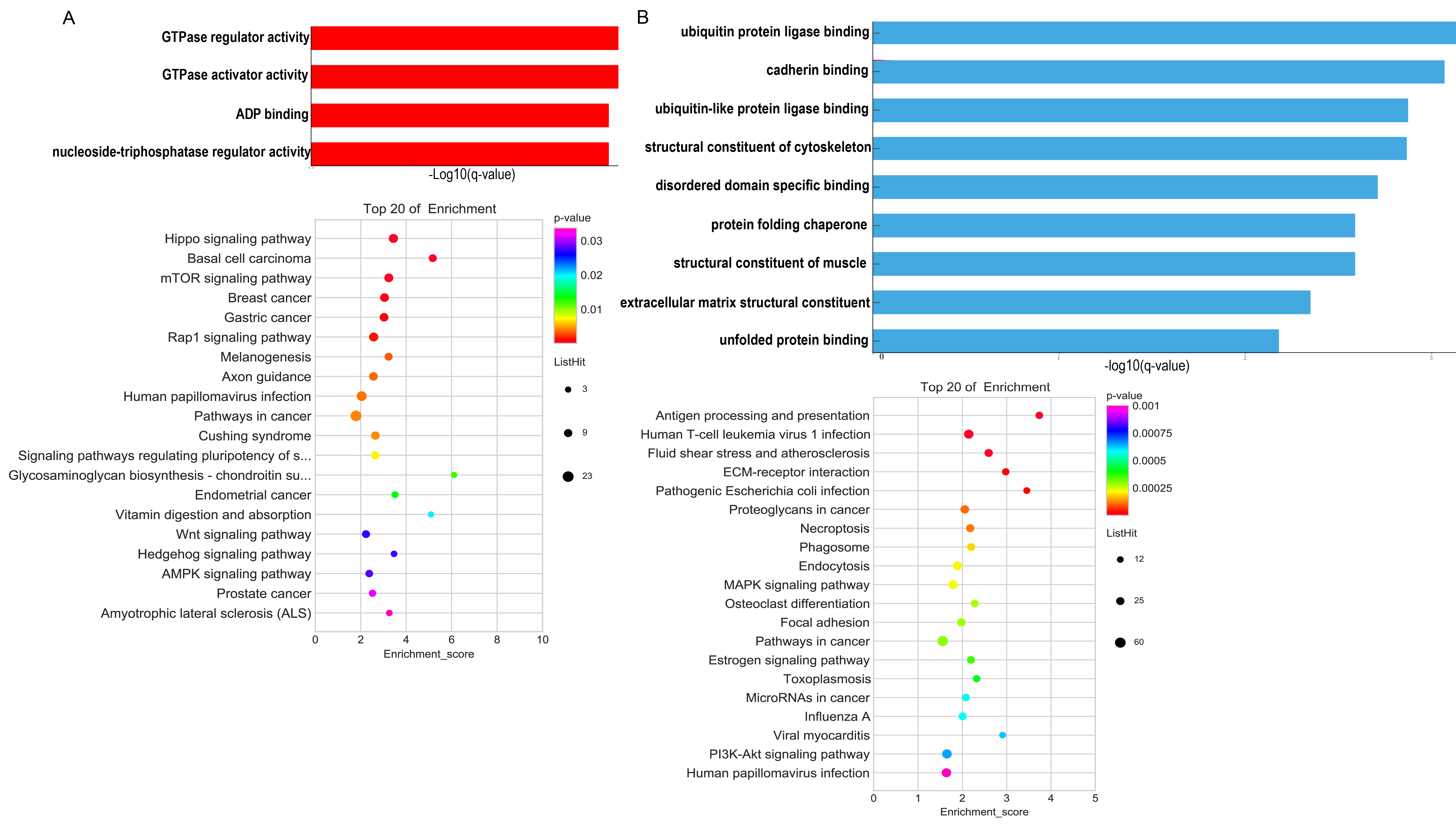

Supplement: Supplementary file 10 — Supporting Information [file CTM2-12-e1006-s003.jpg]

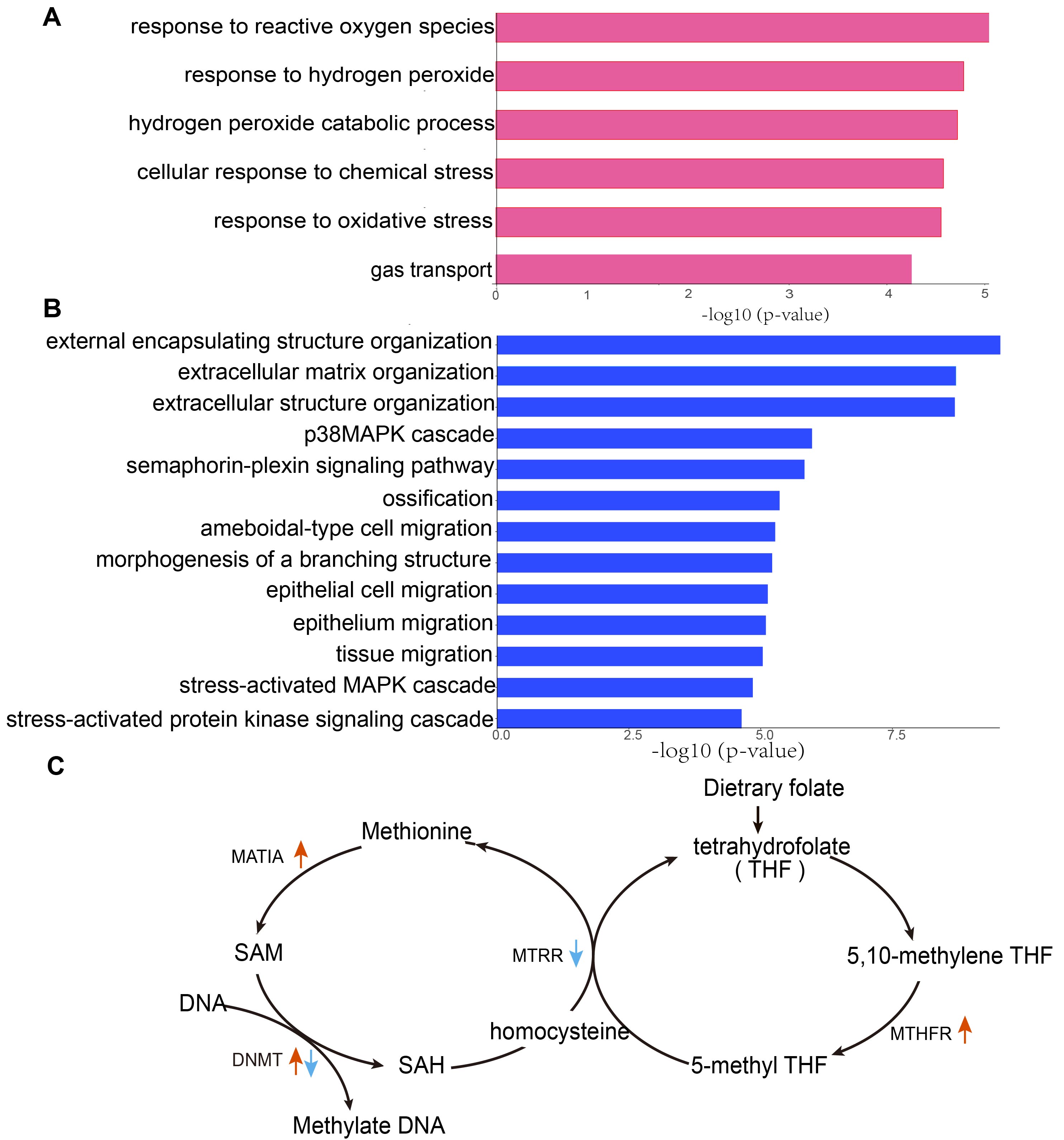

Supplement: Supplementary file 11 — Supporting Information [file CTM2-12-e1006-s010.jpg]

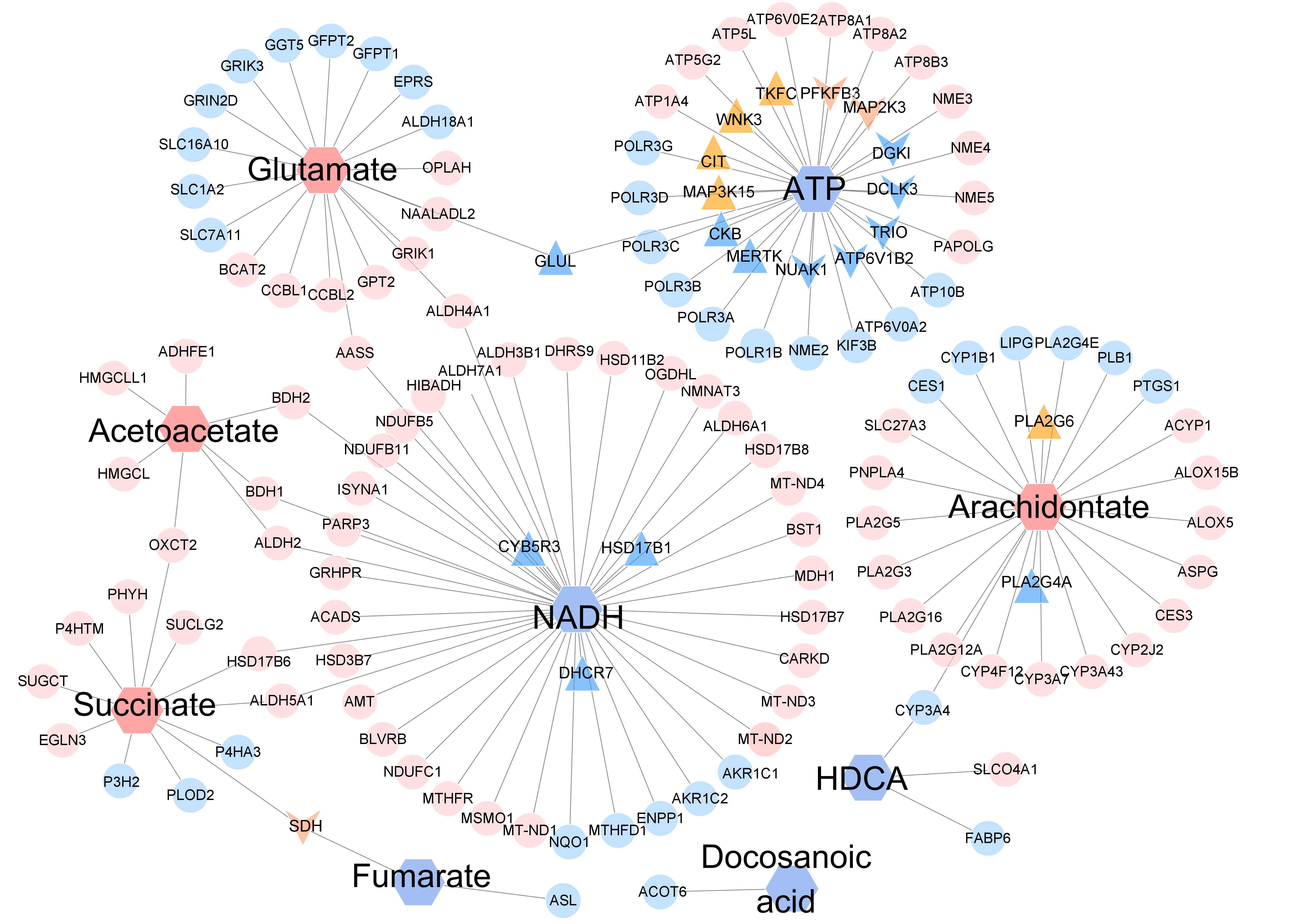

Supplement: Supplementary file 12 — Supporting Information [file CTM2-12-e1006-s007.jpg]
